# Effects of hyperventilation on oxygenation, apnea breaking points, diving response, and spleen contraction during serial static apneas

**DOI:** 10.1007/s00421-023-05202-7

**Published:** 2023-04-15

**Authors:** Frank Pernett, Pontus Bergenhed, Pontus Holmström, Eric Mulder, Erika Schagatay

**Affiliations:** 1 Environmental Physiology Group, Department of Health Sciences, Mid Sweden University, Östersund, Sweden; 2 Swedish Winter Sports Research Centre, Östersund, Sweden

**Keywords:** Hypoxia, Breath-hold diving, Blackout, Hypocapnia

## Abstract

**Purpose:**

Hyperventilation is considered a major risk factor for hypoxic blackout during breath-hold diving, as it delays the apnea breaking point. However, little is known about how it affects oxygenation, the diving response, and spleen contraction during serial breath-holding.

**Methods:**

18 volunteers with little or no experience in freediving performed two series of 5 apneas with cold facial immersion to maximal duration at 2-min intervals. In one series, apnea was preceded by normal breathing and in the other by 15 s of hyperventilation. End-tidal oxygen and end-tidal carbon dioxide were measured before and after every apnea, and peripheral oxygen saturation, heart rate, breathing movements, and skin blood flow were measured continuously. Spleen dimensions were measured every 15 s.

**Results:**

Apnea duration was longer after hyperventilation (133 vs 111 s). Hyperventilation reduced pre-apnea end-tidal CO_2_ (17.4 vs 29.0 mmHg) and post-apnea end-tidal CO_2_ (38.5 vs 40.3 mmHg), and delayed onset of involuntary breathing movements (112 vs 89 s). End-tidal O_2_ after apnea was lower in the hyperventilation trial (83.4 vs 89.4 mmHg) and so was the peripheral oxygen saturation nadir after apnea (90.6 vs 93.6%). During hyperventilation, the nadir peripheral oxygen saturation was lower in the last apnea than in the first (94.0% vs 86.7%). There were no differences in diving response or spleen volume reduction between conditions or across series.

**Conclusions:**

Serial apneas  revealed a previously undescribed aspect of hyperventilation; a progressively increased desaturation across the series, not observed after normal breathing and could heighten the risk of a blackout.

## Introduction

Hypoxic syncope or “blackout” (BO) is a potentially fatal condition associated with breath-hold diving. When fainting underwater, a diver must be immediately rescued by a fellow diver, or they will likely drown. In freediving competitions, this is resolved by the involvement of safety divers, but among recreational divers, such safety systems are often lacking. For example, between 2001 and 2013, 175 breath-hold diving-related deaths were reported in Australia of which 22 cases were considered to be associated with hypoxic BO (Lippmann 2019). The Divers Alert Network (DAN) collected information worldwide from 2004 to 2017, reporting 726 breath-hold diving-associated fatalities mainly in recreational snorkelers, despite not stating which deaths were due to BO. These cases surely are an underrepresentation of the real number of BO-related fatalities (DeWitt et al. 2019).

Single studies have reported a range of risk factors for BO, including extreme bradycardia (Joulia et al. 2013), arrhythmias (Wolf 1964), excess post-exercise oxygen consumption (Lindholm and Gennser 2005), and vascular collapse (Bouten et al. 2020). However, a more widely accepted risk factor is hyperventilation (Craig 1961, 1976; Edmonds and Walker 1999; Lippmann and Pearn 2012) as this breathing pattern reduces the arterial pressure of carbon dioxide (P_a_CO_2_)_._ Lowered P_a_CO_2_ delays the physiological apnea breaking point when an urge to breathe arises, thus increasing apnea duration (Hill 1973; Lin et al. 1974; Bain et al. 2017). If breath-hold duration increases to the point that extreme hypoxia develops, BO may result.

As hypercapnia develops during apnea, and to some degree influenced by hypoxemia (Otis et al. 1984; Breskovic et al. 2012), the ventilatory drive is stimulated, giving rise to involuntary breathing movements (IBM). The start of IBM is considered a hallmark of the physiological breaking point of apnea (Fowler 1954; Agostoni 1963; Hill 1973; Lin et al. 1974). Divided by this point, a maximal effort apnea is characterized by two phases: the “easy phase” and the subsequent “struggle phase” (Dejours 1965). Hyperventilation thus increases the duration of the easy phase by overriding the physiological warning system which may potentially lead to severe hypoxia (Craig 1961; Lindholm and Gennser 2005; Kumar and Ng 2010; Lippmann and Pearn 2012). So far, it is unknown if the risk of BO associated with hyperventilation relies upon the delay of the apnea breaking point per se, or if it affects the depletion of oxygen stores (Sadler et al. 2020), e.g., by affecting the oxygen conserving cardiovascular diving response (Andersson et al. 2002) or the spleen contraction which enhances blood oxygen stores (Schagatay et al. 2001).

Recreational divers have a higher risk of death due to BO (Dunne et al. 2021). These divers do not perform single maximal dives—but typically make serial dives with short pauses. On expert freedivers, it was reported that hyperventilation before static apnea was not associated with an increased risk of BO even under extreme hypoxia (Lindholm and Lundgren 2006). Importantly, most of the studies of hyperventilation effects involve a single dive, and not serial freediving—the most common diving pattern in recreational divers. Professional freedivers like the Ama, who engage in breath-hold diving sessions of 4–5 h with serial dives, rarely report BO (Rahn et al. 1965). The Ama do not use hyperventilation before diving (Hong et al. 1963), while this is common in recreational divers (Craig 1976; Edmonds and Walker 1999; Lippmann and Pearn 2012).

There seems to be no information in the literature on the effects of hyperventilation during serial diving on the risk of BO. We, therefore, aimed to assess the development of hypoxia during a series of static apneas and determine whether hyperventilation before apnea affected oxygenation and apnea breaking points. We also aimed to reveal whether hyperventilation affects the diving response and the splenic contraction, mechanisms potentially protective against hypoxia.

## Methods

### Participants

The study included 18 adult participants (6 females and 12 males) with a mean ± SD age 30 ± 7 years, height 177 ± 8 cm, weight 76 ± 11 kg, and lung vital capacity of 5.4 ± 1.1 L. All participants were healthy and not training breath-hold diving; 15 were classified in class 2 indicating involvement in breath-hold diving during some period in life, but not during the last year, and 3 were in class 1, with no prior experience in breath-hold diving (Schagatay 1996). The participants received written and verbal information about the protocol after which they signed an informed consent. The protocol was approved by the regional human ethics board in Umeå and complied with the Helsinki Declaration of 2004.

### Study design

**Fig. 1:**
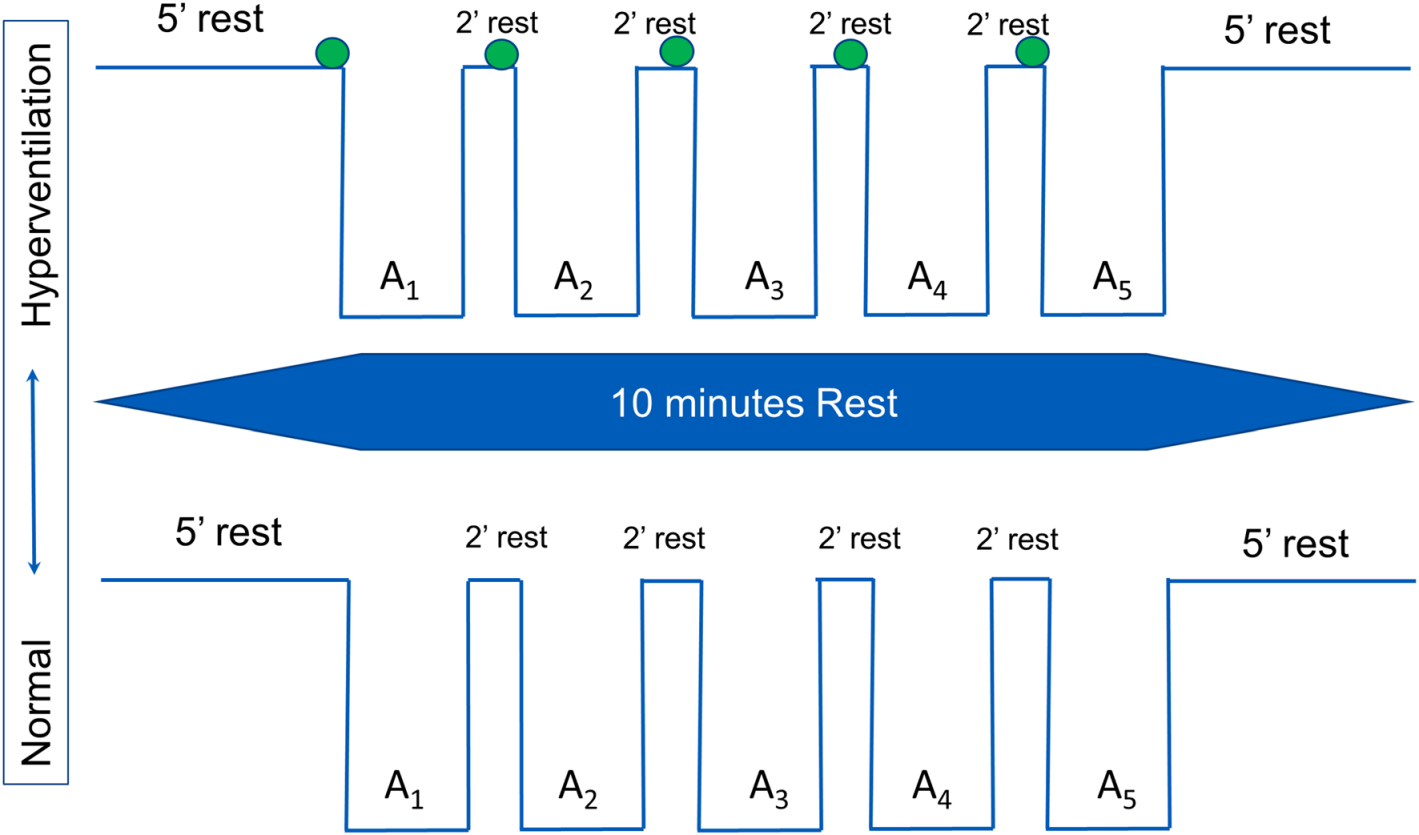
Apnea protocol used for normal breathing (*NB*) and hyperventilation (*HV*) series, which were done in weighted order. The green circle indicates 15 s of hyperventilation

The study evaluated a series of five simulated dives in two conditions: (1) normal breathing before apnea (*NB*) and (2) hyperventilation for 15 s before apnea (*HV*). All the participants were tested in the two conditions during a single visit to the laboratory (Fig. 1). The order of the conditions was weighted between the participants.

### Procedures

Participants arrived at the laboratory after 12 h without any alcohol consumption or strenuous exercise and 2 h without eating or drinking caffeine-containing beverages. Height and weight were measured. Slow vital capacity was measured in triplicate in standing conditions and the largest volume was used (Vitalograph Compact Expert^®^ spirometer, Vitalograph, Buckingham, UK). The participants then rested for 15 min in the prone position while instrumented before the apnea series started. Between apneas, subjects rested prone with the head on a removable pillow covering the water container used for apneic face immersions, with arms on the sides of the container. The participants performed a series of five apneas, with face immersion in cold water (Schagatay and Andersson 1998) to voluntary maximal duration, with 2 min of recovery between apneas (Fig. 1). In the normal breathing condition (*NB*), the participants breathed normally before every apnea, while in the hyperventilation (*HV*) condition, they hyperventilated for 15 s before apnea. The participant was instructed in advance on how to perform the hyperventilation based on a previous protocol (de Bruijn et al. 2008). A researcher made a 2-min countdown before starting. At 30 s before apnea, a nose clip was applied, and 20 s before apnea, a mouthpiece was offered to breathe through. Ten seconds before the apnea, the countdown continued second by second.

**Fig. 2:**
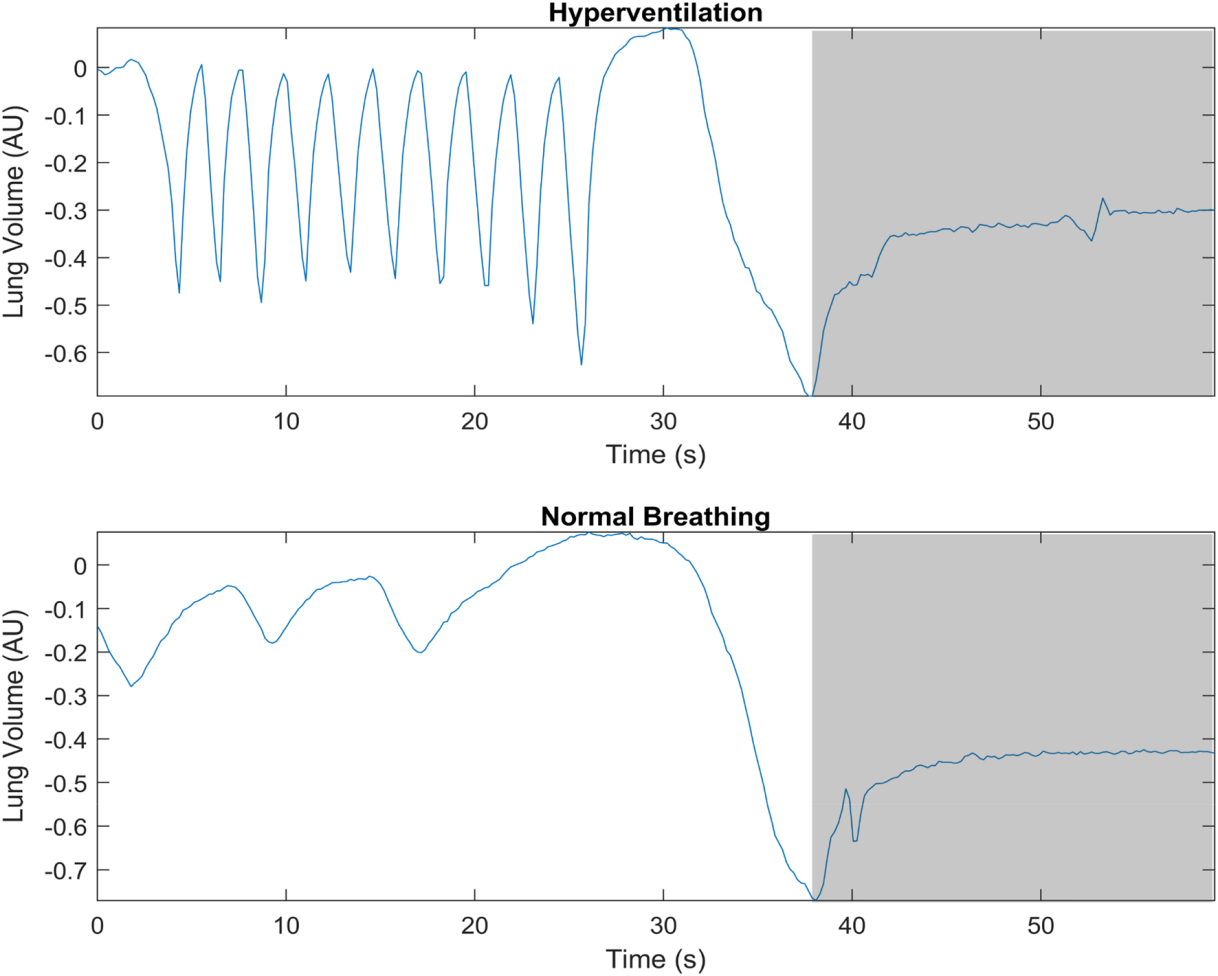
Recordings from the chest bellows show breathing movements in the same participant before the fifth apnea during hyperventilation (top panel) and normal ventilation (bottom panel). The grey areas indicate apneas. AU, arbitrary units

Participants were instructed to exhale completely and make a large but not maximal inhalation before starting the apnea on volition, a technique that results in a volume of around 85% of the vital capacity (Schagatay and Holm 1996; Fig. 2). An experimenter closely monitored SpO_2_ and was ready to interrupt the apnea if it fell below 65%. When recordings of the first condition had ended the participants had a 10-min rest, before baseline recordings for the second condition started, thus the total time between the last apnea in one condition and the first of the other condition was 20 min (Fig. 1). The water temperature for face immersion was 15 ± 1 °C and the room temperature was 23 ± 1 °C. The barometric pressure (*P*_B_) was 762 ± 6 mmHg (102 ± 0.8 kPa).

### Measurements

Continuous variables were recorded from 5 min before apneas until 5 min after the series. Peripheral arterial hemoglobin oxygen saturation (S_p_O_2_) and heart rate (HR) were measured on the second finger or third finger of the right hand via a pulse oximeter (3900, Datex-Ohmeda, Louisville, USA). Respiratory movements were measured via a lab-constructed pneumatic chest bellows connected to an amplifier and an analog recorder (Fig. 2). Skin blood flow (SkBF) was measured via laser-Doppler flowmeter (Periflux 5000, Perimed, Järfälla, Sweden) at the distal phalanx of the right thumb, where the probe was taped after confirming consistent readings. These measurements were recorded simultaneously with a time marker with a data acquisition system (MP160, Biopac Systems, Inc., Goleta, USA).

The fraction of exhaled gases was measured before and after every apnea. Exhaled end-tidal carbon dioxide (E_T_CO_2_) was measured via an infrared-based gas measurement module (CO2100C, Biopac Systems, Inc., Goleta, USA), and exhaled end-tidal oxygen (E_T_O_2_) was measured via a paramagnetic-based gas measurement module (O2100C, Biopac Systems, Inc., Goleta, USA) through a mouthpiece connected to a T-valve with 2 one-way valves (AFT21, Biopac Systems, Goleta, USA) that have a dead space of 80 mL, and a disposable bacterial filter. The partial pressures of the exhaled gases (*P*E_T_CO_2_ and *P*E_T_O_2_) were obtained by multiplying the fraction of the respective gases by the dry *P*_B_ (*P*_B_—saturated water vapor pressure).

Spleen length and thickness were measured on frozen images taken every 15 s from the dorsal side via ultrasonic imaging (MyLab 25 gold equipped with a CA621 probe, Esaote, Genoa, Italy).

### Data analysis

Baseline values used for S_p_O_2_, HR, and SkBF were averages for the 60–30 s before each apnea to minimize effects from the previous apnea and by the anticipation of the next apnea. S_p_O_2_ nadir was defined as the lowest S_p_O_2_ recorded after every apnea.

To assess the initial tachycardia during apnea, we calculated the average heart rate during the first 30 s of apnea (HR_peak_). The “mean HR” during apnea, used to assess the bradycardia, excluded those first 30 s.

SkBF during apnea was defined as the mean value during the whole apnea duration. As the measurements are in perfusion units, percent change during apnea from baseline was used.

The onset of IBM was obtained by visual inspection of the respiratory movements curve and was defined as the first negative deflection after a flat line, which was followed by rhythmical negative deflections.

Spleen sectional area was calculated using length and thickness (Koga and Morlkawa 1975): *S* = 0.8 × *a* × *b*, where *S* is the sectional area, *a* is the length, and *b* is the thickness. Spleen volume was calculated as a function of the sectional area (Koga 1979): *V* = (7.53×S) − 77.56 Splenic volume baseline was defined as the average of the 5 min before the first apnea. Apnea values were defined as initial (first recorded value during apnea), middle (average of the measurements during apnea and final (last recorded value before resuming breathing).

To compare the S_p_O_2_ between conditions with different duration breath holds, the value at the end of apnea in NB was compared with the HV apnea at the same duration.

### Statistical analysis

One outlier was detected in apnea duration, S_p_O_2,_ and *P*E_T_O_2_ during normal breathing, which had a studentized residual value of 3.36. It was considered a genuine value and the results of the analysis of variance (ANOVA) did not change without the outlier, so the participant was kept in the analysis.

The data are reported as mean ± standard deviation (SD). The data were tested for normality using Shapiro–Wilk’s test. A two-factor [time (apnea 1 to apnea 5) vs. group (normal breathing and hyperventilation)] repeated-measures ANOVA was used to compare apnea duration, *P*E_T_O_2_, *P*E_T_CO_2_, SpO_2_, HR, IBM-onset, and splenic contraction. When a two-way interaction was found, a one-way ANOVA was run to evaluate the simple main effects of conditions and apnea. A Bonferroni correction was applied for multiple comparisons. Effect sizes were estimated by the partial eta squared (${\eta }_{p}^{2}$) and the generalized eta squared (${\eta }_{G}^{2}$) and are presented with a 90% confidence interval (CI). An effect size of 0.01–0.05 was considered small, from 0.06 to 0.13 was considered medium and from and above 0.14 was considered large (Cohen 1988; Bakeman 2005; Lakens 2013). Significance was accepted at *p* < 0.05.

End-tidal gas variables (*P*E_T_O_2_ and *P*E_T_CO_2_) were lost for five participants due to dysfunction of the gas measurement modules; therefore, that analysis is made on 13 participants. Spleen measurements were collected in all participants, but due to technical failure of the ultrasonic machine, part of the recorded data was lost, and the complete data include 11 participants. The number of participants included is indicated in the results.

## Results

All participants completed the apnea protocol successfully. The studied variables changed equally between sexes in response to apnea and hyperventilation, and therefore, men and women were analyzed as one single group.

### Apnea duration

**Fig. 3:**
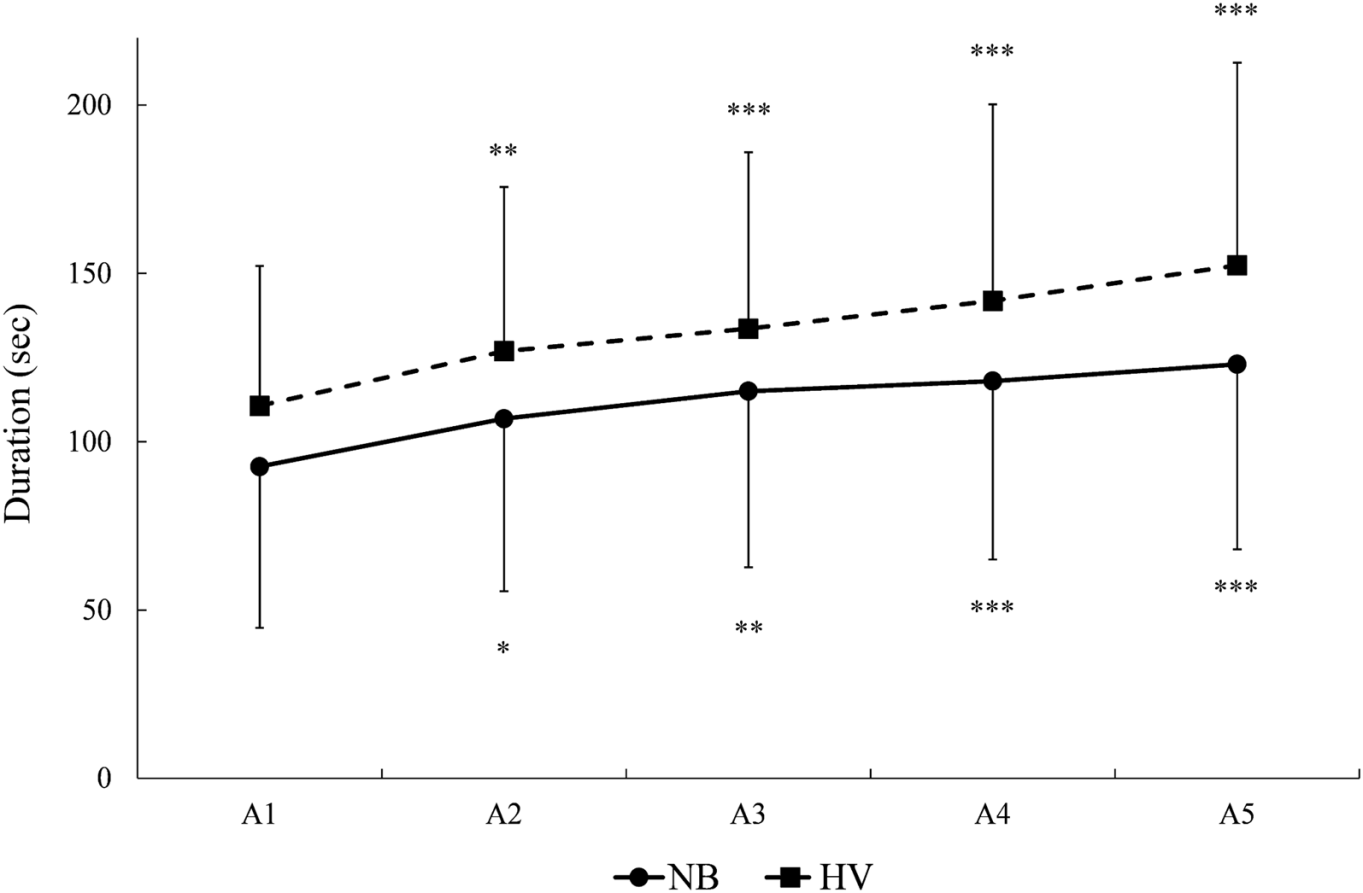
Mean apnea duration for apnea 1–5 (A1–A5), with SD bars, for the two conditions: normal breathing (NB) and hyperventilation (HV). *p < 0.05, **p < 0.01 and ***p < 0.001 compared with A1 in the same condition (*n* = 18)

Mean (SD) apnea duration was longer in *HV*, at 133 ± 6 s, compared with 111 ± 12 s in *NB* (*p* < 0.001, ${\eta }_{p}^{2}$ = 0.61, 90% CI [0.30–0.73], ${\eta }_{G}^{2}$ = 0.05; Table 1, Fig. 3). There was an increase in duration across apnea A1–A5 in both conditions (*p* < 0.001, ${\eta }_{p}^{2}$ = 0.70, 90% CI [0.50–0.78], ${\eta }_{G}^{2}$ = 0.06; Table 1, Fig. 3).

**Table 1:** Apnea duration between conditions and within apnea series

	Normal breathing (s)	Hyperventilation (s)	Time difference (s)	Time increase (%)
A1	93 ± 48	111 ± 42	18	19
A2	107 ± 51^†^	127 ± 49 ^†*^	20	19
A3	115 ± 52^†^	134 ± 52^†*^	19	16
A4	118 ± 53^†^	142 ± 58^†*^	24	20
A5	123 ± 55^†^	152 ± 60^†*^	29	24
Mean	111 ± 12	133 ± 6^*^	22	20

Mean (SD) apnea duration values in seconds for A1–A5. Mean, an average of the five apneas in the same condition

*Significantly different from *NB*; ^†^significantly different from A1 in the same condition. (*n* = 18)

### End-tidal carbon dioxide

**Fig. 4:**
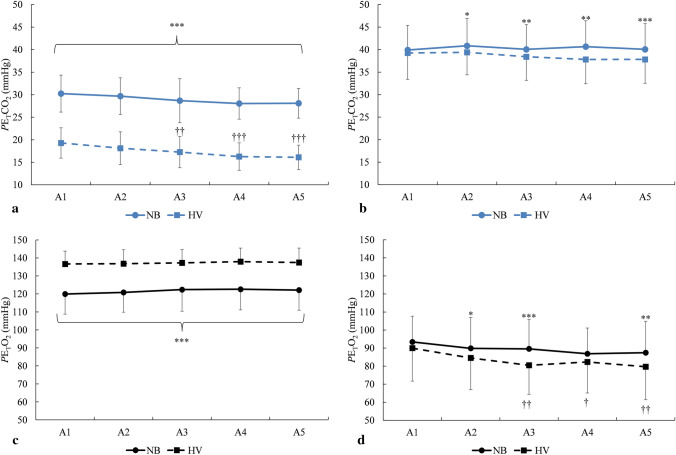
End-tidal carbon dioxide pressures (*P*E_T_CO_2_) at **a** the start of each apnea 1–5 and **b** at the end of apnea 1–5 in the two breathing conditions. End-tidal oxygen pressures (*P*E_T_O_2_) at **c** the start of each apnea 1–5 and **d** at the end of apnea 1–5 in the two conditions. *p < 0.05, **p < 0.01, and ***p < 0.001 between conditions; ^†^*p* < 0.05, ^††^*p* < 0.01, and ^†††^*p* < 0.001 compared with A1 in the same condition (*n* = 13)

At the start of each apnea in *HV,* the mean *P*E_T_CO_2_ was 17.4 ± 3.4 mmHg (2.3 ± 0.5 kPa) which was lower than the 29.0 ± 4.0 mmHg (3.9 ± 0.5 kPa) in *NB* (*p* < 0.001, ${\eta }_{p}^{2}$ = 0.93, 90% CI [0.86–0.96], ${\eta }_{G}^{2}$ = 0.76; Fig. 4a, Fig. 6). In both conditions, *P*E_T_CO_2_ at the start was lower in the last apnea compared with the first (*p* = 0.001, ${\eta }_{p}^{2}$ = 0.53, 90% CI [0.32–0.61], ${\eta }_{G}^{2}$ = 0.11; Fig. 4a).

At the end of apneas in *HV,* the mean *P*E_T_CO_2_ was lower in *HV* with 38.5 ± 5.2 mmHg (5.1 ± 0.7 kPa), compared to after *NB* with 40.3 ± 5.5 mmHg (5.4 ± 0.7 kPa, *p* = 0.005, ${\eta }_{p}^{2}$ = 0.49, 90% CI [0.12–0.67], ${\eta }_{G}^{2}$ = 0.03; Fig. 4b, Fig. 6). *P*E_T_CO_2_ at the end of apnea was similar across both series (*p* = 0.272, ${\eta }_{p}^{2}$ = 0.10, 90% CI [0.00–0.25], ${\eta }_{G}^{2}$ = 0.006; Fig. 4b).

### Involuntary breathing movements

**Fig. 5:**
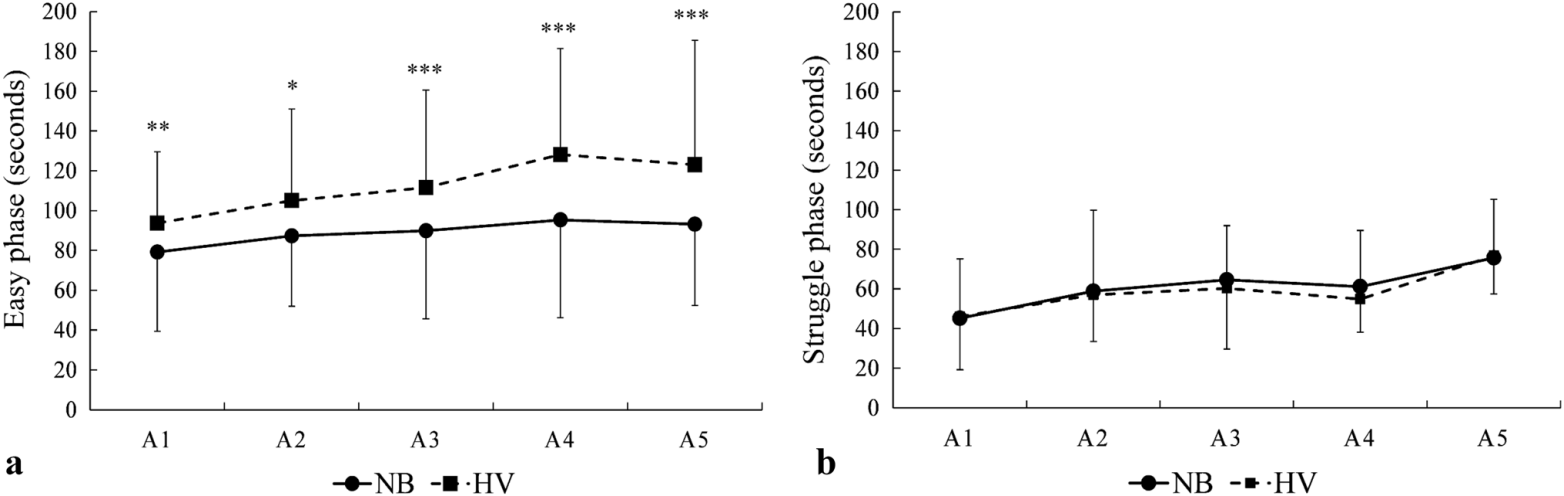
Duration of the easy (**a**) and struggle (**b**) phases for apneas 1–5 (A1–A5) after normal breathing (*NB*) and hyperventilation (*HV*) for the seven subjects with involuntary breathing movements (IBM) in all apneas. ***p < 0.001, **p < 0.01, and *p < 0.05 between conditions

Of the 18 participants, 7 (39%) had IBM in A1–A5 in both conditions, allowing comparison of easy phase duration. The start of the struggle phase was delayed in *HV*, at a mean of 112 ± 49 s, compared with 89 ± 42 s in *NB* (*p* = 0.003, ${\eta }_{p}^{2}$ = 0.79, 90% CI [0.32–0.88], ${\eta }_{G}^{2}$ = 0.08; Fig. 5). There was no difference across the series in any condition (*p* = 0.446, ${\eta }_{p}^{2}$ = 0.34, 90% CI [0.02–0.46], ${\eta }_{G}^{2}$ = 0.04; Fig. 5).

### End-tidal oxygen

**Fig. 6:**
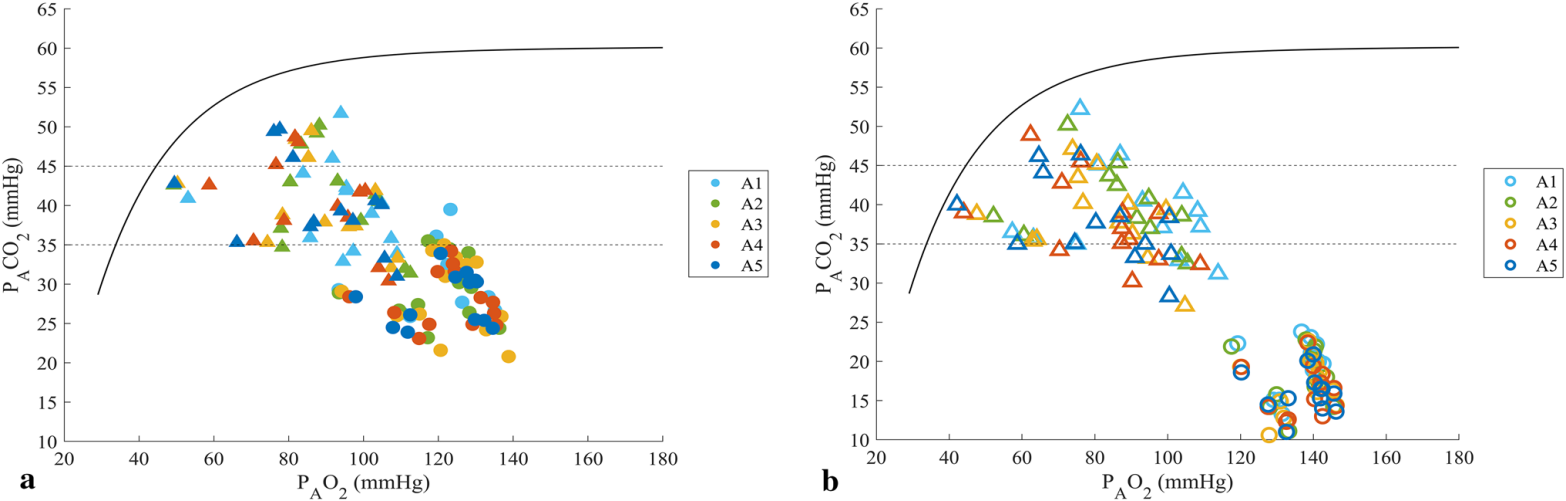
O_2_–CO_2_ diagram showing individual alveolar values during *NB* (**a**) and *HV* (**b**). Circles represent values at the start of apnea. Triangles represent values at the end of apnea. Filled symbols indicate *NB* (**a**) and empty symbols indicate *HV* (**b**). Colors show apnea series. Horizontal dashed lines show the range of normal P_A_CO_2_. Black continuous lines represent the conventional apnea breaking point (Agostoni 1963) (*n* = 13)

At the start of each apnea, *P*E_T_O_2_ was higher in *HV* with 137.2 ± 7.4 mmHg (18.0 ± 1.0 kPa) compared with 121.6 ± 11.1 mmHg (16.2 ± 1.5 kPa) in *NB* (*p* < 0.001, ${\eta }_{p}^{2}$ = 0.917, 90% CI [0.80–0.94], ${\eta }_{G}^{2}$ = 0.43; Fig. 4c, Fig. 6). There was no difference across A1–A5 in any condition (*p* = 0.094, ${\eta }_{p}^{2}$ = 0.25, 90% CI [0.05–0.35], ${\eta }_{G}^{2}$ = 0.009; Fig. 4c).

At the end of each apnea, *P*E_T_O_2_ was lower in *HV* with a mean of 83.4 ± 17.3 mmHg (11.1 ± 2.3 kPa), compared with 89.4 ± 15.6 mmHg (11.9 ± 2.1 kPa) in *NB* (*p* = 0.005, ${\eta }_{p}^{2}$ = 0.498, 90% CI [0.12–0.68], ${\eta }_{G}^{2}$ = 0.04; Fig. 4d, Fig. 6). The *P*E_T_O_2_ at the end of apnea was also decreasing across the series in *HV*, being higher in A1 than in A5 (*p* < 0.001, ${\eta }_{p}^{2}$ = 0.426, 90% CI [0.16–0.55], ${\eta }_{G}^{2}$ = 0.03; Fig. 4d).

### Peripheral oxygen saturation

**Fig. 7:**
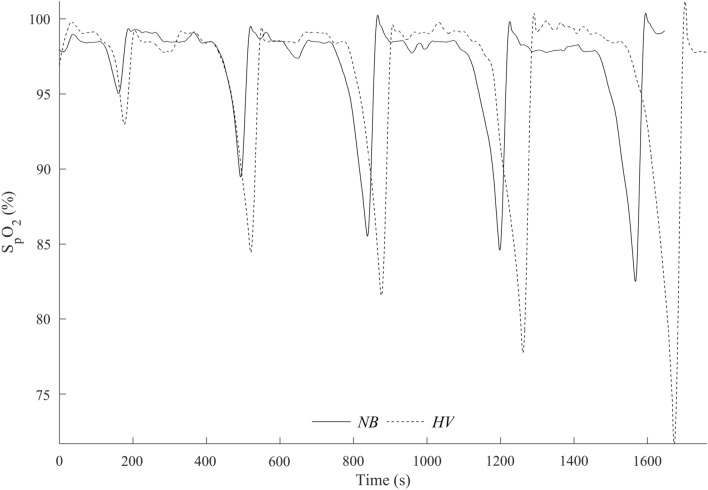
S_p_O_2_ recording in one participant during series of five dives after normal breathing (*NB*) and hyperventilation (*HV*)

Figure 7 shows a representative recording of SpO_2_ across the NB and HV series from one subject, where the *HV* series results in progressive desaturation.

**Fig. 8:**
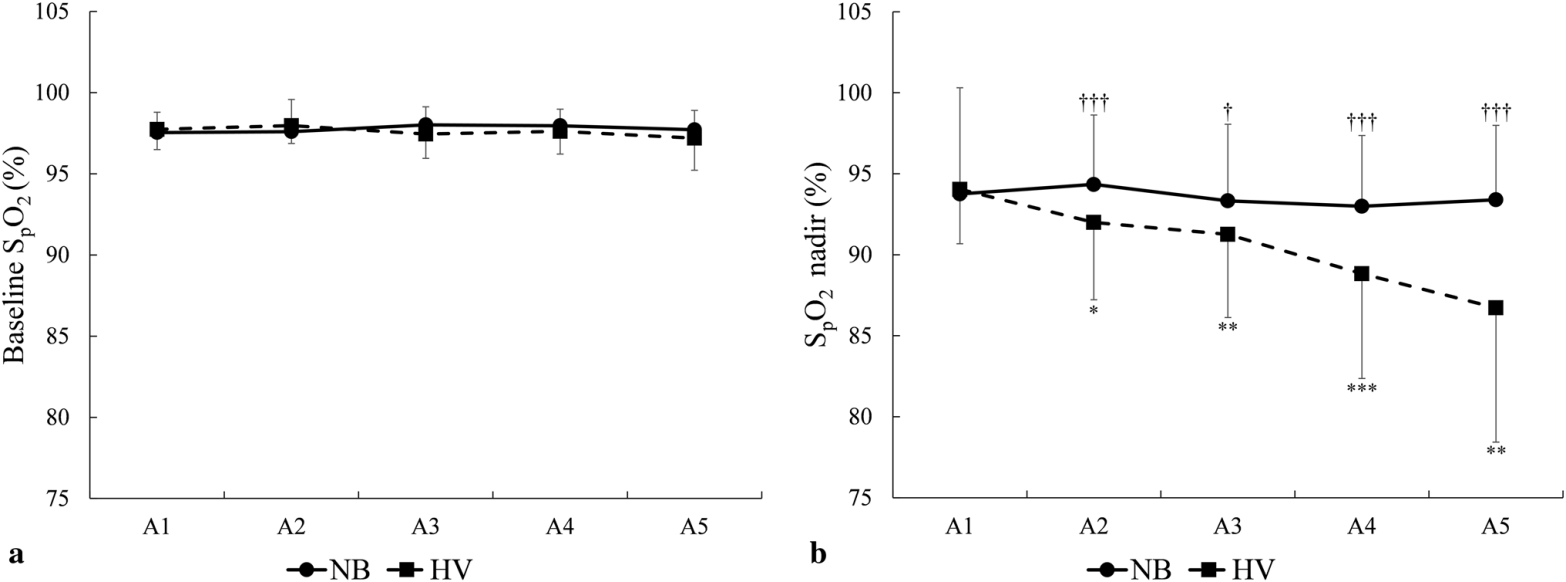
Mean baseline pre-apnea SpO_2_ from A1–A5 (**a**) and at post-apnea nadir (**b**)*.* *p < 0.05, **p < 0.01, ***p < 0.001 compared with A1 in the same condition; ^†^*p* < 0.05, ^††^*p* < 0.01, ^†††^*p* < 0.001 compared with HV (*n* = 18)

**Fig. 9:**
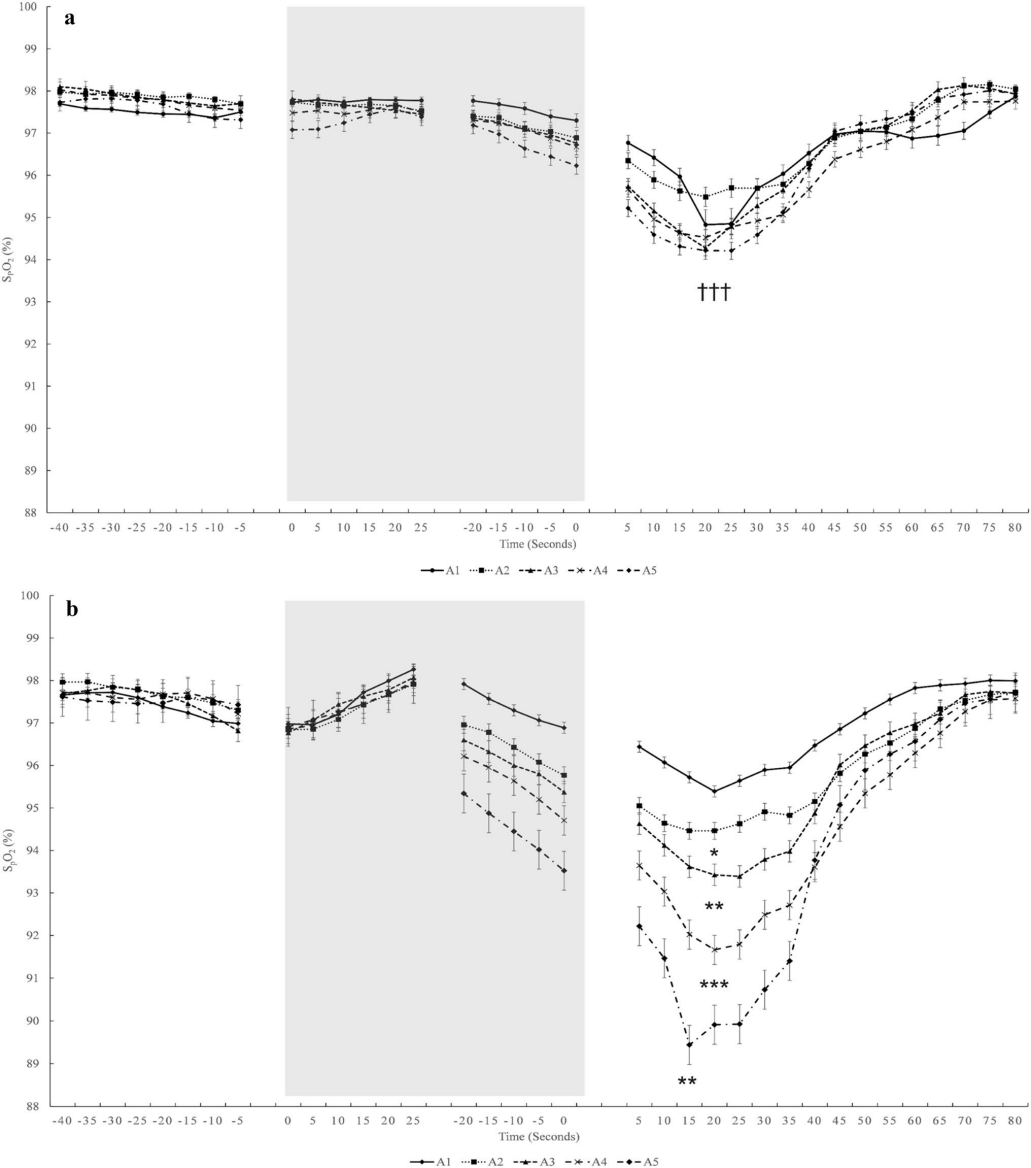
Mean (SE) SpO_2_ in *NB* (**a**) and *HV* (**b**) series during A1–A5. The grey boxes indicate apnea time. Negative time values before apnea time indicate breathing just before apneas. Positive values after apnea time indicate recovery time directly after apneas and partly overlap with pre-apnea values of the subsequent apnea. The gap in the middle of apnea time has variable duration, as apnea duration was different between participants. *p < 0.05, **p < 0.01, ***p < 0.001 indicates significance compared with A1 in the same condition; ^†††^*p* < 0.001 indicates significance compared with *HV* (*n* = 18)

Mean baseline S_p_O_2_ was similar between conditions and apnea series (*p* = 0.421, ${\eta }_{p}^{2}$ = 0.04, 90% CI [0.00–0.24], ${\eta }_{G}^{2}$ = 0.01; Fig. 8a). S_p_O_2_ nadir did not change across the apnea series in *NB*, (*p* = 0.576, ${\eta }_{p}^{2}$ = 0.03, 90% CI [0.00–0.14], ${\eta }_{G}^{2}$ = 0.10, Fig. 8b, Fig. 9a), but it decreased during the apnea series in *HV* (Fig. 8b, Fig. 9b) with the lowest value in A5 (*p* < 0.001, ${\eta }_{p}^{2}$ = 0.48, 90% CI [0.25–0.61], ${\eta }_{G}^{2}$ = 0.17, Fig. 9b). Mean S_p_O_2_ nadir was lower in *HV* with 90.6 ± 5.6%, compared with *NB* with 93.6 ± 4.9% (*p* < 0.001, ${\eta }_{p}^{2}$ = 0.644, 90% CI [0.35–0.76], ${\eta }_{G}^{2}$ = 0.10; Fig. 8b). In 4 participants, the S_p_O_2_ nadir resulting from the fifth apnea during *HV* was ≤ 75%. There was no difference between *NB* and *HV* when comparing the S_p_O_2_ values at the same time point as the average in *NB* at the end of apnea was 96.4 ± 3.5% and in *HV* was 96.0 ± 3.5% (*p* = 0.25, ${\eta }_{p}^{2}$ = 0.08, 90% CI [0.00–0.23], ${\eta }_{G}^{2}$ = 0.003).

### Diving response

**Fig. 10:**
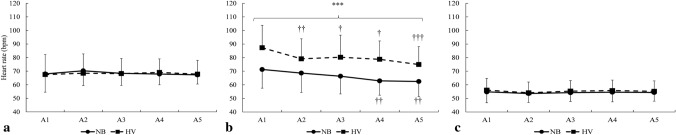
Heart rate average for baseline (**a**), HR_peak_ (**b**), and during apnea (**c**). ***p < 0.001 between normal (NB) and hyperventilation (HV) conditions; ^†††^*p* < 0.001, ^††^*p* < 0.01, and ^†^*p* < 0.05 compared with A1 in the same condition

The HR at baseline was similar in both conditions, with a mean of 68 ± 12 bpm in *NB* and 68 ± 9 bpm in *HV* (*p* = 0.923, ${\eta }_{p}^{2}$ = 0.001, 90% CI [0.00–0.04], ${\eta }_{G}^{2}$ = 0.0001; Fig. 10a). Recovery HR between apneas was also similar within series (*p* = 0.795, ${\eta }_{p}^{2}$ = 0.02, 90% CI [0.00–0.15], ${\eta }_{G}^{2}$ = 0.004; Fig. 10a).

**Fig. 11:**
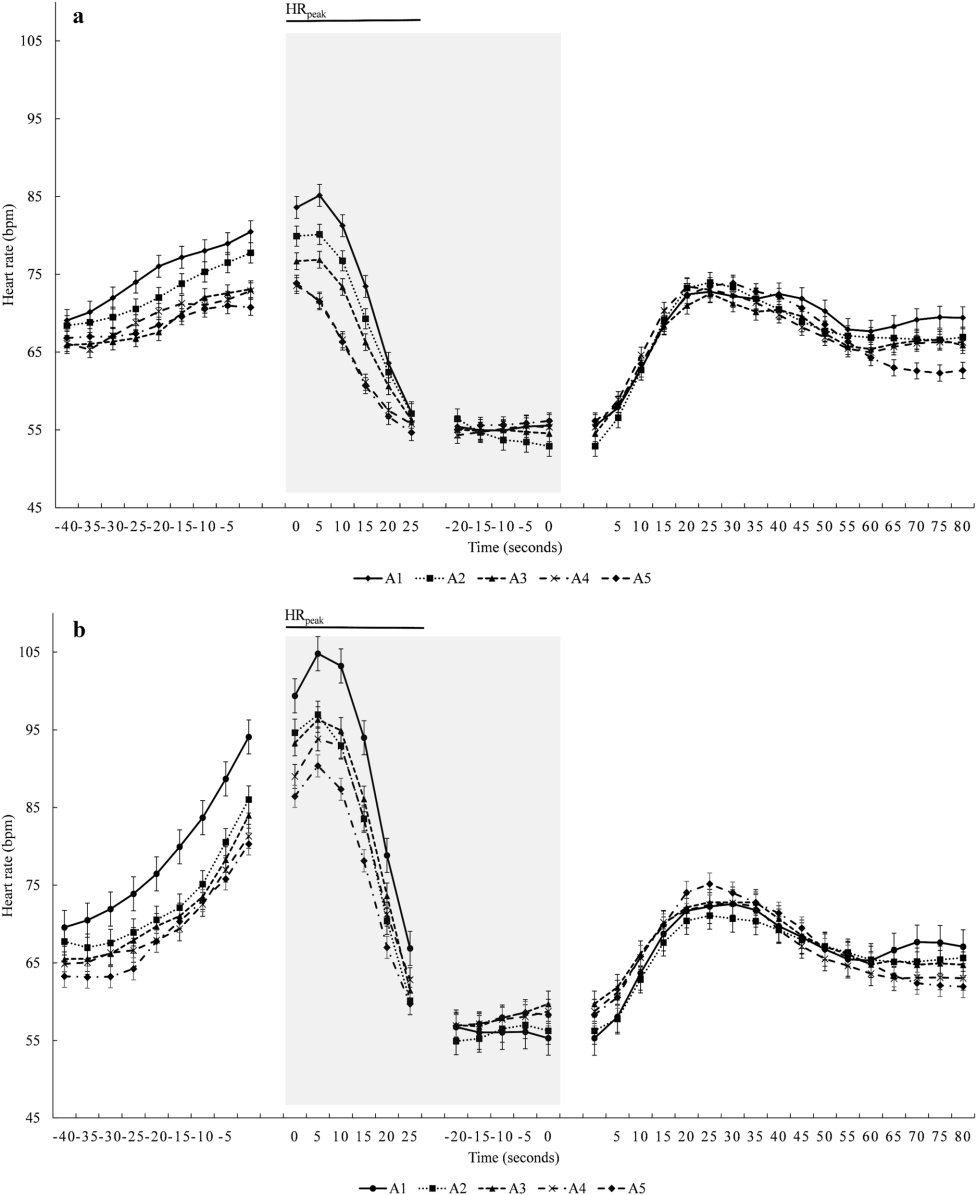
Mean (SE) heart rate during apnea 1–5 at Normal (**a**) and HV (**b**). The grey boxes indicate apnea time. Negative time values before apnea time indicate breathing. Positive values after apnea time indicate recovery time and overlap with the previous values of the subsequent apnea. The gap in the middle of apnea time has variable duration, as apnea time was different between participants (*n* = 18)

Mean HR_peak_ after apnea onset was higher in *HV* with 80 ± 15 bpm compared with 66 ± 13 bpm in *NB* (*p* < 0.001, ${\eta }_{p}^{2}$ = 0.76, 90% CI [0.54–0.84], ${\eta }_{G}^{2}$ = 0.23; Figs. 10b, 11b). During the *NB* series, HR_peak_ fell from 71 ± 14 bpm in A1 to 62 ± 11 bpm in A5 (*p* < 0.001, ${\eta }_{p}^{2}$ = 0.39, 90% CI [0.21–0.48], ${\eta }_{G}^{2}$ = 0.07; Figs. 10b, 11a). Across the *HV* series, HR_peak_ fell from A1, at 87 ± 17 bpm, to A5 at 75 ± 13 bpm, (*p* < 0.001, ${\eta }_{p}^{2}$ = 0.36, 90% CI [0.18–0.45], ${\eta }_{G}^{2}$ = 0.07; Figs. 10b, 11b).

The mean HR during apnea in *NB* was 54 ± 7 bpm and in *HV* was 55 ± 8 bpm, hence no differences (*p* = 0.205, ${\eta }_{p}^{2}$ = 0.09, 90% CI [0.00–0.32], ${\eta }_{G}^{2}$ = 0.01; Fig. 10c). Neither were there any differences in mean HR during apnea across the series (*p* = 0.232, ${\eta }_{p}^{2}$ = 0.08, 90% CI [0.00–0.19], ${\eta }_{G}^{2}$ = 0.01; Fig. 10c). The HR reduction in percent from baseline was also the same at 19% in *NB* and 18% in *HV* (*p* = 0.694, ${\eta }_{p}^{2}$ = 0.09, 90% CI [0.00–0.17], ${\eta }_{G}^{2}$ = 0.003).

**Fig. 12:**
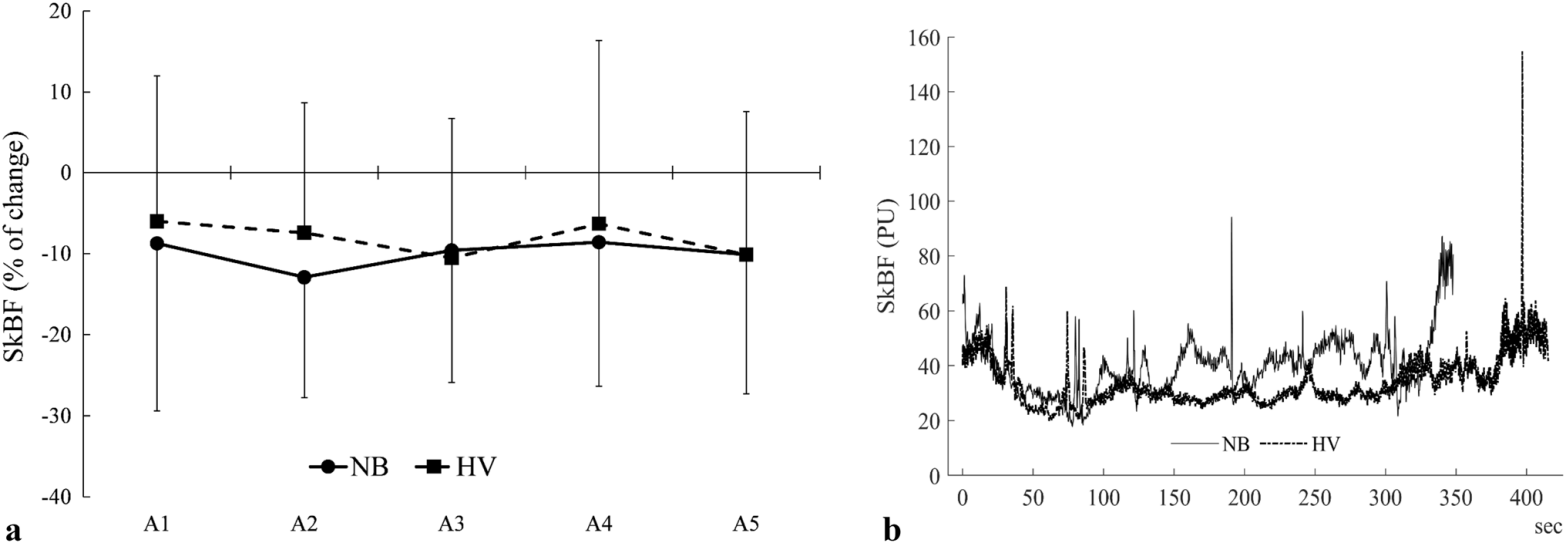
SkBF reduction in percentage from baseline in both conditions from apnea 1 to apnea 5 (**a**) and recordings from one participant in the fifth apnea comparing both conditions (**b**). PU, perfusion units. (*n* = 18)

There was no difference in the SkBF reduction between *NB* with − 10.0 ± 17.3% and *HV* with − 8.1 ± 18.3% (*p* = 0.10, ${\eta }_{p}^{2}$ = 0.10, 90% CI [0.00–0.33], ${\eta }_{G}^{2}$ = 0.01; Fig. 12a). Neither were any differences observed between series (*p* = 0.716, ${\eta }_{p}^{2}$ = 0.02, 90% CI [0.00–0.10], ${\eta }_{G}^{2}$ = 0.01; Fig. 12a).

### Spleen contraction

**Fig. 13:**
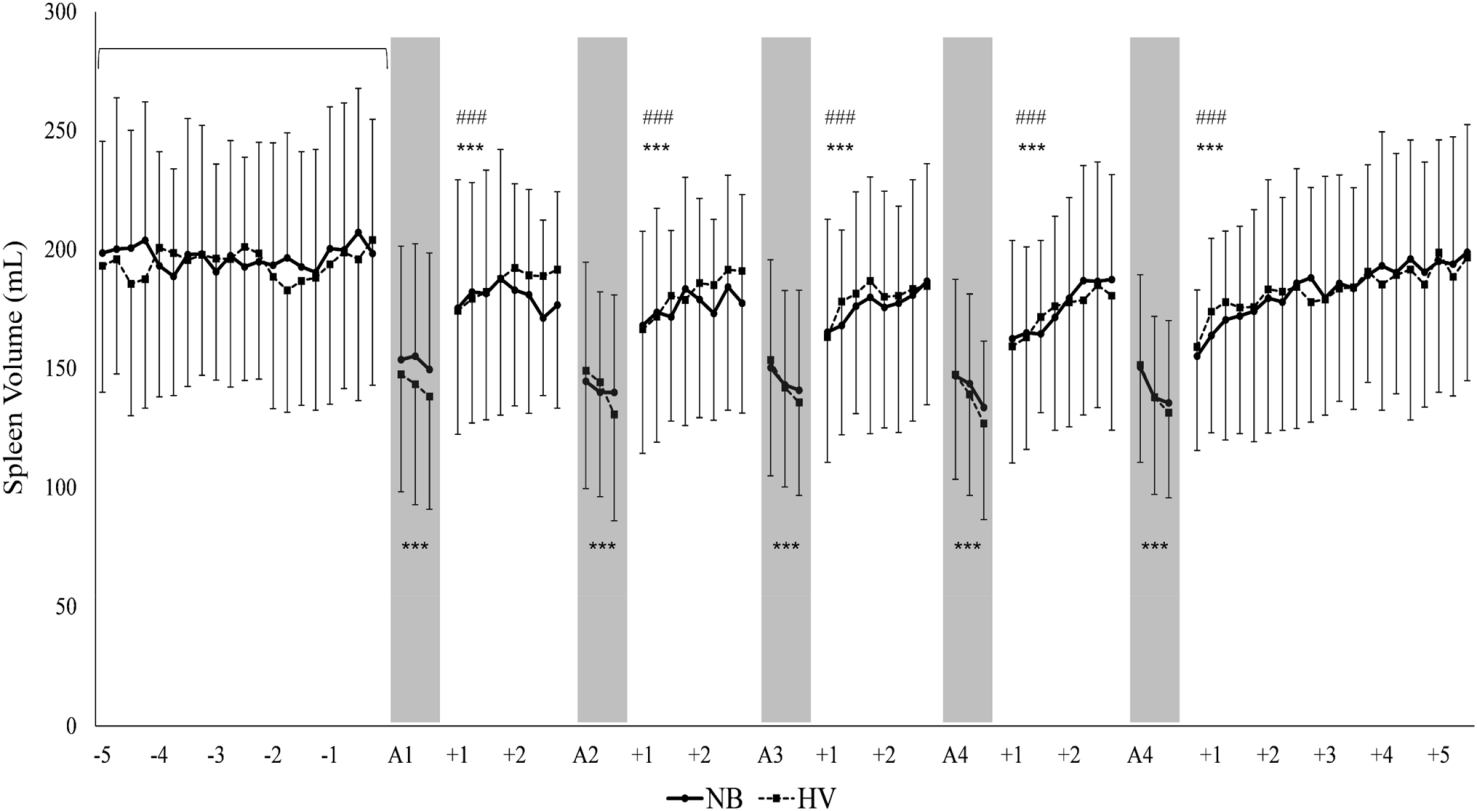
Mean spleen volume during the NB and HV apnea series. The grey boxes indicate apneas. Negative values before A1 indicate baseline measurements 5 min before starting. Positive values after every apnea indicate recovery time in minutes. The brackets indicate that an average period was compared. ***p < 0.001 compared with baseline; ^###^*p* < 0.001 compared with apnea values

Mean (SD) baseline spleen volume before the apnea series was similar at 197 ± 54 mL in *NB* and 194 ± 56 mL in *HV* (*p* = 0.276; Fig. 13). The spleen contracted during all apneas in both *NB* and *HV* series and increased in size during breathing intervals, although not reaching baseline values. The average volume during recovery after every apnea was still 11% lower than baseline in *NB*, and 8% lower in *HV*, from A2 to A5 (*p* = 0.012; Fig. 13).

There were no differences in mean splenic volume reductions during apnea between conditions or within series. During apneas, mean spleen volume was 131 ± 40 mL in NB and 126 ± 43 mL in HV series (*p* = 0.348), reflecting reductions from baseline by 33% and 35%, respectively (*p* < 0.001; Fig. 13). The mean spleen volume during the 2 min of recovery was similar at 176 ± 46 mL in NB and 179 ± 57 mL in HV series, and after 5 min recovery spleen volume was restored to normal in both series (Fig. 13).

## Discussion

Hyperventilation compared to normal ventilation delayed the apnea breaking point and augmented the decrease in *P*E_T_O_2_ and S_p_O_2_, which confirms conclusions from earlier studies that hyperventilation increases the risk of severe hypoxia (Craig 1961, 1976; Lanphier and Rahn 1963; Hill 1973; Lippmann 2019). A novel finding was that the serial apneas with hyperventilation lead to a progressively increased desaturation across the series, which resulted in more pronounced hypoxia in the fifth apnea, involving the development of severe hypoxia in several subjects. This finding could help explain why recreational divers are especially prone to BO, as they typically make a series of dives with short pauses, and slight hyperventilation is common in this group (Craig 1976; Edmonds and Walker 1999; Lippmann and Pearn 2012). They risk to, without notice, passing into the anaerobic phase of the dive, leading to the progressive development of oxygen debt.

This finding could be of great importance during serial diving as an increased oxygen debt would avert a sustainable diving session. This could reflect an inability of recreational divers to pace their diving efficiently. As a comparison, professional Ama divers can do more than 100 dives per dive session with pauses of similar duration as dives, apparently avoiding in a natural way reaching the aerobic dive limit (ADL), where anaerobic metabolism is required, and hence an oxygen debt develops (Hong et al. 1963, 1991; Radermacher et al. 1992; Mohri et al. 1995; Schagatay et al. 2011).

The increased desaturation in the *HV* did not happen during *NB* and apnea duration could explain this difference. *NB* apneas were shorter than *HV* apneas, but the apnea duration increased across the series in both conditions. The oxygen–hemoglobin dissociation curve does not change dramatically at the beginning of apnea, and therefore, shorter apneas do not lead to severe hypoxemia, while longer apneas are related to much greater hypoxemia per unit of time as the changes in S_p_O_2_ occur in the steep part of the curve. In *HV*, the extra work induced by spontaneous hyperventilation could contribute to an increased oxygen cost as the work of breathing (*W*_breathing_) is higher than increased ventilation due to hypercapnia (Otis 1954).

In *HV*, we also found an increase in HR in the first 30 s of apnea. This finding had been described before after spontaneous hyperventilation, especially when tidal volume is increased, via reduced parasympathetic activity, and possibly, by the pumping effect of the large swings on pleural pressure that increase cardiac preload (Cummin et al. 1986). Additionally, we speculate on another mechanism that links the increased HR at the beginning of apnea with hypoxemia. As spontaneous hyperventilation increases *W*_breathing_ (Cummin et al. 1986; Coast and Krause 1993), it could increase oxygen consumption ($\dot{V}{O}_{2}$). As minute ventilation was not measured, it is difficult to calculate the increased *W*_breathing_. If the apnea is started with a high $\dot{V}{O}_{2}$, the oxygen stores would decrease faster. In a series of apneas, this could induce mixed venous desaturation that persists even after resuming breathing (Sands et al. 2010) and the following apnea could start with an oxygen debt. Despite starting with higher *P*E_T_O_2_ values in HV, values were lower at the end of apnea compared to NB, but with no changes during the series of apneas. This finding could be explained by the measurement place, as it reflects changes in the alveolar pressure of oxygen (P_A_O_2_), while S_p_O_2_ is affected by local metabolism (Ellsworth et al. 2009), and as the P_A_O_2_ pathway during apnea is curvilinear, shorter apneas relate to higher values (Taboni et al. 2020). Regardless of the increase in P_A_O_2_ during hyperventilation and the effect it has on P_a_O_2_, the S_p_O_2_ was lower during *HV* and was of the same value when comparing same duration apneas. This emphasizes the lack of benefit of hyperventilation and could reflect the cost of using accessory expiratory muscles.

### Apnea breaking points

Our model with 15 s of hyperventilation reached an average *P*E_T_CO_2_ value of 17 mmHg, which is similar to other models that used 30 s (Landsberg 1974), 2 min (Craig 1961), and spontaneous duration of hyperventilation (Otis et al. 1984; Lindholm and Lundgren 2006). This finding shows that even short-period hyperventilation can induce significant hypocapnia and subsequent prolongation of apnea with the risk of developing severe hypoxia. As *P*E_T_CO_2_ was lower at the end of hyperventilation apneas, it is evident that hypoxia contributes to triggering the respiratory drive.

The fact that our participants had limited experience in breath-hold diving could explain why IBM occurred only in seven participants, as many may have stopped at the first urge to breathe (Schagatay et al. 2000). Indeed, the longer apnea duration in *HV* was associated with an increase in the easy phase duration, clearly showing the effect of hyperventilation on the physiological breaking point. While many participants had a dive duration within the easy phase, the participants that surpassed this point are apparently at greater risk for developing dangerous hypoxia. In competition divers, increased sensitivity to hypoxia per se could be protective (Andersson and Schagatay 2009). The relative importance of hypercapnia and hypoxia, respectively, to the development of an urge to breathe should be further studied in the novice compared to trained divers for a better understanding of risk.

We did not find any difference in the diving response (bradycardia and vasoconstriction) between the breathing conditions or across the series of apneas. The HR_peak_ was lower in the last apneas in both conditions, which is likely related to more relaxation toward the end of the series. The reduction in SkBF displayed a great variation among participants, showing that the sympathetic response to apnea is highly individual (Busch et al. 2019), but there were no systematic differences between breathing conditions or across the series. As none of these main factors comprising the diving response were affected by hyperventilation, it can be concluded that the protective mechanism of the diving response is not enhanced to counteract the lower oxygen delivery during longer apneas.

Another studied protective response during apnea was spleen contraction, which elevates the circulating amount of erythrocytes resulting in enhanced oxygen stores, and has previously been associated with longer apneas (Schagatay et al. 2001, 2005, 2012; Baković et al. 2003). The spleen volume reduction during apnea in the present study was significant in both conditions, and the spleen did not return to baseline volume between the series of apneas. However, we did not find any differences in spleen volumes between normal breathing and hyperventilation or across the series of apneas, showing that neither did this protective mechanism counteract the enhanced desaturation with prolonged apneic duration. In most previous studies, spleen volumes were only measured between apneas (Hurford et al. 1990; Espersen et al. 2002; Baković et al. 2003; Schagatay et al. 2005, 2012; Prommer et al. 2007; Richardson et al. 2012; Elia et al. 2021) or during short-duration apneas (Palada et al. 2007), while we measured it during longer apneas. It is further interesting to note that this response, known to be initiated by hypoxia but enhanced by hypercapnia (Richardson et al. 2012), was similar in both conditions, and we suggest that this could be due to a balancing out between series by enhanced desaturation and reduced hypercapnia in *HV*.

Our study shows that even a short period of hyperventilation delays the physiological breaking point, prolongs apnea duration, and increases desaturation versus normal breathing. This effect increases progressively across the hyperventilation series, and a long series of dives could potentially lead to SpO_2_ levels associated with hypoxic BO. There is no change in the hypoxia-protective cardiovascular diving response or spleen contraction to compensate for this effect. The risk with hyperventilation appears multifactorial and includes a greater drop in S_p_O_2_ due to prolonged apnea duration, delayed apnea breaking point, and no changes in protective mechanisms. If the serial effect with progressively increased desaturation seen during hyperventilation could be due to incomplete central venous oxygenation between long dives despite normalized S_p_O_2_, requires further investigation.

As the experiment was conducted during static apnea, we avoided the increased metabolic demands from exercise associated with swimming dives, and thus, our conclusions may be limited to static apnea. Further studies are needed during repeated swimming dives to reveal if this imposes the same risk of BO, although we speculate that the enhanced metabolic rate with exercise would aggravate the situation, as oxygen depletion would be faster.

## Conclusion

Serial dives revealed a previously undescribed aspect of hyperventilation; a progressively increased desaturation across the series, which was not observed after normal breathing. The fifth dive with hyperventilation resulted in desaturation below 75% in four subjects. This observation could help explain why hyperventilation increases the risk of BO, particularly in recreational divers, who typically perform serial dives.

The risk with hyperventilation appears multifactorial and includes a greater drop in S_p_O_2_ across a series of apneas. A monitoring system of S_p_O_2_ in freedivers could be developed to alert divers when serial dives lead to an increased desaturation and give a signal that a longer resting pause should be taken.

## Data Availability

Data will be made available upon reasonable request.

## Abbreviations

${\eta }_{G}^{2}$: Generalized eta squared
${\eta }_{p}^{2}$: Partial eta squared
ADL: Aerobic dive limit
ANOVA: Analysis of variance
BO: Blackout
CI: Confidence interval
DAN: Divers alert network
*P*E_T_CO_2_: End-tidal carbon dioxide pressure
*P*E_T_O_2_: End-tidal oxygen pressure
HR: Heart rate
HV: Hyperventilation
IBM: Involuntary breathing movements
kPa: Kilopascal
mL: Milliliters
NB: Normal breathing
P_a_CO_2_: Arterial pressure of carbon dioxide
P_A_CO_2_: Alveolar pressure of carbon dioxide
P_A_O_2_: Alveolar pressure of oxygen
*P*_B_: Barometric pressure
SD: Standard deviation
SE: Standard error
SkBF: Skin blood flow
S_p_O_2_: Peripheral oxygen saturation
*V*O_2_: Oxygen consumption
*W*_breathing_: Work of breathing
